# BRAF mutant colorectal cancer: ErbB2 expression levels as predictive factor for the response to combined BRAF/ErbB inhibitors

**DOI:** 10.1186/s12885-020-6586-0

**Published:** 2020-02-17

**Authors:** Evelina Miele, Luana Abballe, Gian Paolo Spinelli, Zein Mersini Besharat, Giuseppina Catanzaro, Martina Chiacchiarini, Alessandra Vacca, Agnese Po, Carlo Capalbo, Elisabetta Ferretti

**Affiliations:** 1 Department of Molecular Medicine, Sapienza University of Rome, Viale Regina Elena 291, 00161 Rome, Italy; 2 Center for Life NanoScience@Sapienza, Istituto Italiano di Tecnologia, 00161 Rome, Italy; 3 Present address: Department of Onco-Haematology, Cellular and Genetic Therapy of Pediatric Tumors, Bambino Gesù Children’s Hospital, Piazza S. Onofrio, 4, 00165 Rome, Italy; 4 Department of Experimental Medicine, Sapienza University of Rome, Viale Regina Elena 324, 00161 Rome, Italy; 5 Department of Medical-Surgical Sciences and Biotechnologies, Sapienza University of Rome, Corso della Repubbica, 04100 Latina, Italy; 6 UOC, Territorial Oncology District 1 - ASL Latina, Via Giustiniano snc, 04011 Aprilia, LT Italy; 7 Neuromed Institute, Località Camerelle, 86077 Pozzilli, IS Italy; 8 Istituto Pasteur Italia - Fondazione Cenci Bolognetti, Sapienza Università di Roma, Viale Regina Elena 291, 00161 Rome, Italy

**Keywords:** BRAFV^600E^ mutation, Colon cancer, Molecular therapy, Afatinib, Vemurafenib, ErbB2

## Abstract

**Background:**

Colorectal cancer (CRC) is a heterogeneous disease with a complex biology and a wide number of altered genes such as BRAF, KRAS and PIK3CA. Advances with new-targeted therapies have been achieved and available treating options have prolonged patient’s survival. However, BRAF-mutated CRC patients remain unresponsive to available therapies with RAF inhibitors (RAFi) alone or combined with ErbB inhibitors (ErbBi). These unmet needs require further exploitation of oncogenic signaling in order to set up individualized treatments.

**Methods:**

To this end, we tested the efficacy of single agent or combined treatments using the BRAFi, vemurafenib and two different ErbBi: panitumumab and afatinib in CRC cells characterized by different molecular phenotypes.

**Results:**

Combination strategies with BRAFi and ErbBi achieved a better response in BRAF^V600E^ mutated cells expressing high levels of ErbB2.

**Conclusions:**

Our findings support the importance of ErbB2 evaluation in BRAF-mutated CRC patients and its role as a positive predictor factor of response to BRAFi/ErbBi combination.

## Background

Colorectal carcinoma (CRC) is the third leading cause of death from cancer worldwide [1]. Recent studies have shed light on the complex biology of CRC, which reflects its heterogeneous genetic background with alterations involving a variety of genes (e.g., TP53, EGFR, RAS, PIK3CA, BRAF, and PTEN). These efforts have provided fundamental information for the development of novel treatments targeting key signaling pathways in the tumor [2]. Advances made in the diagnosis and treatment of CRC have undoubtedly prolonged patient survival. However, unfavorable outcomes are still the rule for certain CRCs that are particularly aggressive [3, 4]. Up to 10% of metastatic CRCs are BRAF V600E-mutated, and display a worse prognosis [3, 5]. These tumors are relatively unresponsive to currently used chemotherapy protocols [6, 7], and in these cases, second-line treatment with anti-epidermal growth factor receptor (EGFR)-antibodies is rarely beneficial [8–10].

BRAF (along with CRAF and ARAF) belongs to a family of genes that encode serine–threonine kinases, which heterodimerize and are activated by several receptor tyrosine kinases (RTKs), such as EGFR. Together with downstream MEK and ERK proteins, RAF kinases constitute a powerful mitogen-activated protein kinase (MAPK) pathway [3, 4]. The BRAF mutations found in CRC usually occur at the V600 hotspot and lead to constitutive activation of BRAF^V600E^, which signals as a monomer in the absence of upstream signaling through the RAS signaling pathway. This explains at least in part the resistance of BRAF^V600E^ CRCs to EGFR pathway blockade [11, 3].

Selective inhibitors of RAF kinases, such as vemurafenib, regorafenib [12], and dabrafenib [13], have recently been introduced into clinical practice, and BRAF inhibitors have proved to be highly effective for the treatment of BRAF-mutant melanoma. In contrast, when used alone, these drugs produce only minimal benefits in BRAF-mutant colorectal cancer [14] because they cause reactivation of RAS (via a feedback mechanism) [15–17] or activation of signaling through the PI3K/AKT pathway [18].

This issue has been addressed in several preclinical and clinical studies, which indicate that EGFR blockade and BRAF targeting act synergistically to inhibit ERK pathway signaling in BRAF^V600E^ mutant colon cancers [3, 16, 19]. However, in a recent study, the efficacy of targeted therapies with RAF inhibitors or combinations with MEK and EGFR inhibitors were found to vary widely in cohorts of patients with BRAF^V600E^ CRCs [20]. The therapeutic failure of BRAF inhibition in these cases might be related to other mutations and/or the activation of other signaling pathways. It is becoming increasingly clear that the biology of BRAF mutant patients varies widely and this heterogeneity must be taken into account for the development of effective targeted therapies [20].

In the study described below, we tested the efficacies of BRAF and EGFR inhibitors, alone and in combination, against BRAF^V600E^ mutant colon cancer cell lines with different mutational profiles (Table 1). Based on the results that emerged, we propose the use of these drugs in the treatment of patients with BRAF-mutant CRCs, based on the tumor’s expression of ErbB2.

**Table 1:** Gene mutation profiles of the colon cancer cell lines used in this study

Cell Line	RAS	BRAF	PI3KCA	EGFR	TP53	APC	SMAD4	other
SW-48	wt	wt	wt	c.2155G > A p.G719S	wt	wt	wt	CTNNB1 c.98C > A p.S33Y
								FBXW7 c.2001delG p.S668 fs*39
Colo205	wt	c.1799 T > A	wt	wt	c.308_333 > TA	c.4666_4667insA	c.1_667del667	–
		p.V600E			p.Y103_L111 > L	p.T1556 fs*3		
HT-29	wt	c.1799 T > A p.V600E	c.1345C > A p.P449T	wt	c.818G > A R273H	c.2557G > T p.E853* c.4666_4667insA p.T1556 fs*3	c.931C > T p.Q311*	–

## Methods

### Cell lines and reagents

SW-48 (EGFR mutant) (ATCC CCL-231), Colo-205 (BRAF mutant, TP53 mutant, APC mutant) (ATCC CCL-222) and HT-29 (BRAF mutant, PIK3CA mutant) (ATCC HBT-38) human colorectal cancer cells (Table 1) were obtained from the American Type Culture Collection (ATCC). Colo-205 was grown in in RPMI-1640 (supplemented with 10% (v/v) fetal bovine serum, 1% (v/v) penicillin (50Uml-1) - streptomycin (50Uml-1) and 2 mM L-glutamine) and the other ones in Dulbecco’s Modified Eagle’s Medium - high glucose D6429 (supplemented with 10% (v/v) fetal bovine serum, 1% (v/v) penicillin (50Uml-1) - streptomycin (50Uml-1) and 2 mM L-glutamine) confirmed free of mycoplasma contamination by regular testing with PCR Mycoplasma Detection Kit (ABM, Cat. No. G238).

### Inhibitors

The selective BRAF inhibitor vemurafenib and the dual TKI inhibitor afatinib (BIBW2992) were both purchased from SelleckChem (Cat. No. S1267-10MM/1ML and Cat. No. S1011, respectively). Each was dissolved in DMSO to obtain a 10-mM stock solution. Panitumumab (Vectibix®, Amgen, Thousand Oaks, CA) was kindly provided by GPS in a stock solution of 20 mg/ml. (Table 2)

**Table 2:** Biomolecular drugs tested in the study

Drug	Target	Dosage
Vemurafenib (Vem)	BRAF V600E	3 μM
Panitumumab (Pan)	EGFR wt	8 μg/mL
Afatinib (Afa)	TKI (EGFR wt/mut and ERBB2[HER2-Neu])	0.01, 0.1, 1, 10 μM

### Cell viability and cell death assays

Cell viability was evaluated after 48 h of treatment by MTS assay (CellTiter 96® AQueous One Solution Reagent assay, Promega) and cell count by Trypan blue exclusion. Each sample was measured in triplicate and repeated at least three times.

The dye trypan blue exclusion test was used to determine the number of viable or death cells present in a cell suspension (Warren Strober APPENDIX 3B Trypan Blue Exclusion Test of Cell Viability, Current Protocols in Immunology, 2001).

### Western blot assay

Protein lysates were prepared using RIPA buffer (50 mM Tris-HCl, pH 7.6, 0.5% deoxycholic acid sodium salt, 140 mM NaCl, 1% NP-40, 5 mM EDTA, 100 mM NaF, 2 mM Na pyrophosphate) with fresh protease inhibitors (Roche). Lysates were separated on 6% SDS polyacrylamide gels. Proteins were transferred to nitrocellulose membranes (0.45 μm) (PerkinElmer). Membranes were blocked for 1 h at room temperature in 5% nonfat dry milk and incubated overnight at 4 °C with the following antibodies: rabbit anti-human ErbB2 (HER2-Neu) (clone A0485, Dako), rabbit anti-HSP70 (sc-33,575; Santa Cruz Biotechnology). HRP-conjugated secondary antibodies (Santa Cruz Biotechnology) were used in combination with enhanced chemiluminescence (ECL Amersham). Densitometry calculations were made with Image-J software (rsb.info.nih.gov), after verification of non-saturation and background subtraction. Values are expressed as the integrals of each band normalized to weakest band and a ratio between target protein and calibrator proteins (housekeeping–HK: HSP70) is shown.

### Statistical analysis

Results are expressed as means ± s.d. from an appropriate number of experiments.

Statistical analysis was performed using GraphPad Prism software Version 6.0 (La Jolla, CA, USA). Two-way ANOVA test for multiple comparison was used unless otherwise specified.

## Results

### BRAF^V600E^ CRC cell lines are sensitive to the selective BRAF inhibitor vemurafenib and insensitive to the EGFR-inhibitor panitumumab

For our investigations, we selected colorectal carcinoma cell lines according to their mutational status (Table 1). In particular, mutations of the EGFR signaling pathway were considered, in the order of the following molecules: EGFR, RAS (including K-RAS, H-RAS, N-RAS), BRAF, TP53, and PI3K.

Two (Colo205, HT-29) harbored the BRAF^V600E^ mutation with wild-type EGFR; the third (SW-48) was BRAF wild-type (wt) with an EGFR mutation.

### Both BRAF^V600E^ CRC cell lines are sensitive to the selective BRAF inhibitor vemurafenib and insensitive to the EGFR-inhibitor panitumumab in vitro

**Fig. 1:**
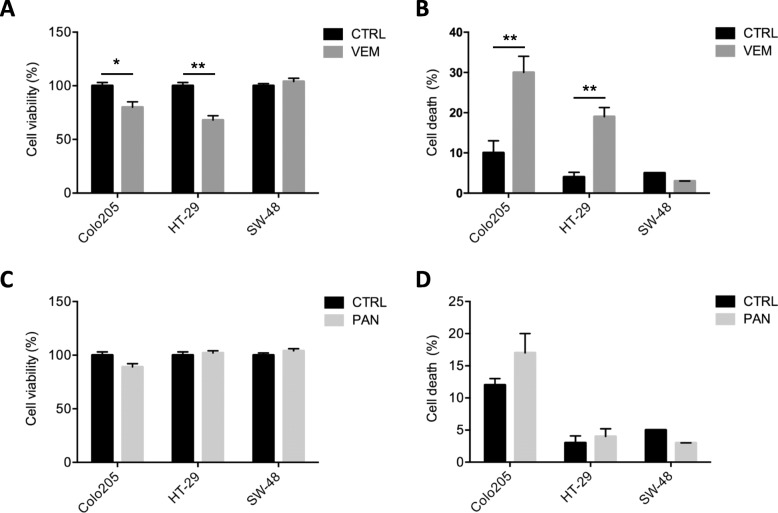
BRAF^V600E^ CRC cell lines are sensitive to VEM but not to PAN. BRAF-mutant and wildtype cells were exposed to (**a**, **b**) vemurafenib (VEM – final concentration 3 μM) or (**c**, **d**) panitumumab (PAN – final concentration 8 μg/mL) for 48 h, harvested, and assayed for cell viability (MTS assay) and cell death (trypan blue staining). Results shown are means ± SD of three independent experiments. Statistics (unpaired t-test with equal SD): *p < 0.05, **p < 0.01 vs. CTRL (untreated cells)

To explore their in vitro sensitivity to BRAF blockade, we first treated the three CRC cell lines with the selective BRAF^V600E^ inhibitor, vemurafenib (VEM). As expected, the BRAF wt SW-48 cells were unaffected by the drug (Fig. 1a and b). In contrast, the BRAF^V600E^ lines, Colo205 and HT-29, both displayed significantly reduced viability and increased death rates after exposure to VEM, which is consistent with previous reports [18]. To investigate the in vitro sensitivity to EGFR blockade, the cell lines were then exposed to panitumumab (PAN), a fully human recombinant monoclonal IgG2 antibody highly specific against EGFR/ErbB-1/HER1. PAN is indicated for the treatment of metastatic CRC with wild-type KRAS [21]. As shown in Fig. 1c and d, however, PAN had no significant growth-limiting effects in any of the cell lines tested, regardless of their EGFR status.

### Co-exposure to panitumumab may enhance the efficacy of vemurafenib in some BRAF^V600E^ CRC cells

Since a synergy between EGFR and BRAF^V600E^ inhibition was previously demonstrated **[**3**,**^11^**]**, we investigated the possible combined action of vemurafenib and panitumumab in cell models tested.

**Fig. 2:**
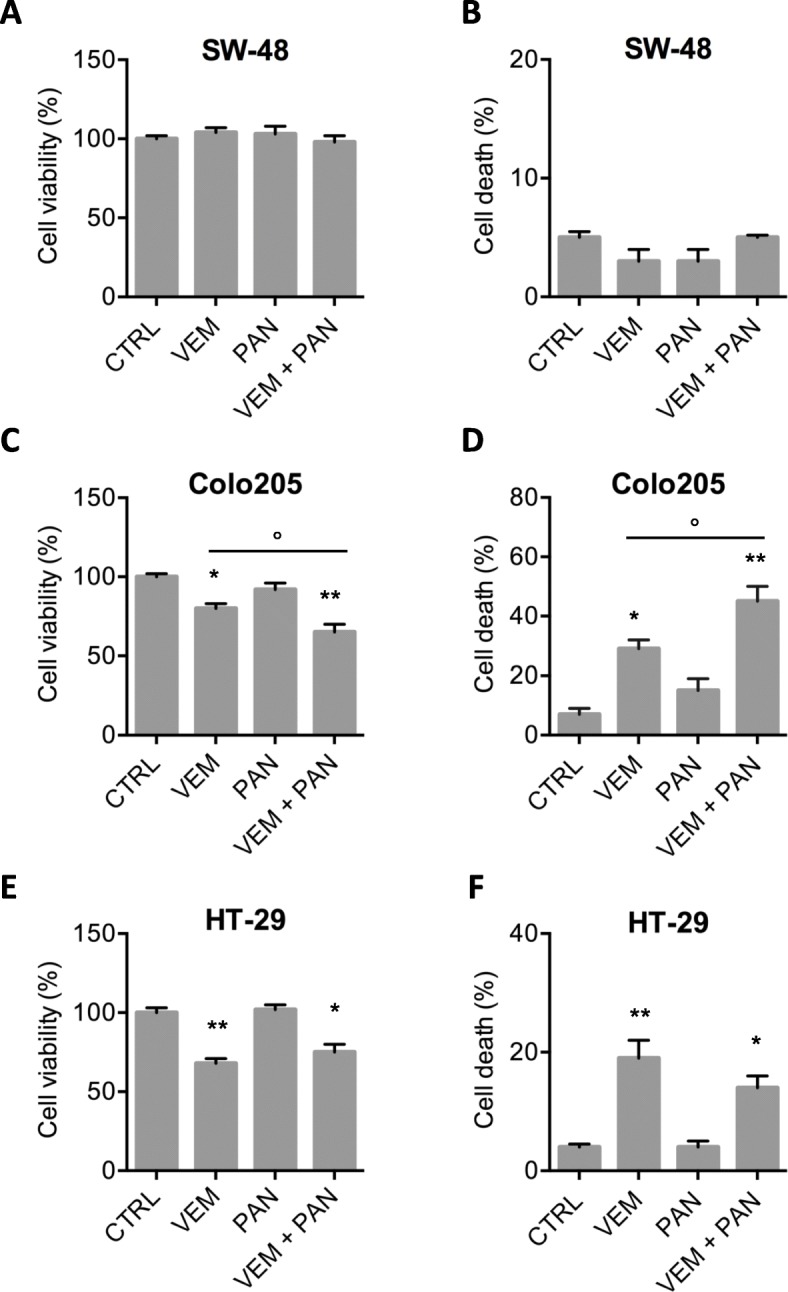
Co-exposure to panitumumab may enhance the efficacy of vemurafenib in some BRAF^V600E^ CRC cells. **a**-**f**. Cell viability (**a**, **c**, **e**) and cell death (**b**, **d**, **f**) assessment in BRAF^V600E^ mutant cells Colo205 and HT-29 and in SW-48 BRAFwt (EGFRmut) cell line, 48 h after the administration of 3 μM vemurafenib (VEM), or 8 μg/mL panitumumab (PAN) or the combination of the two (Vem + Pan). Histograms represent mean ± SD of three independent experiments *p < 0.05, **p < 0.01 vs CTRL; ° p < 0.05 vs VEM

As shown in Fig. 2a and b, vemurafenib and panitumumab, administered singly or combined, had no effects on cell viability or cell death rates in the BRAF-wt/EGFR-mutant SW-48 line. As for the BRAF^V600E^ CRC cells, the two drugs exerted synergic growth-limiting effects in the Colo205 line (Fig. 2c and d). In contrast, in HT-29 cells, the efficacy of the combination was not significantly different from that of vemurafenib alone (Fig. 2e and f).

Our results from data presented in Figs. 1 and 2 show that the combined targeting of EFGR and BRAF^V600E^ can be beneficial in BRAF^V600E^ cells.

### BRAF^V600E^ CRC cells respond to different doses of the pan-ErbB-family inhibitor afatinib

We then proceeded to explore whether a molecule with a broader action on ErbB family receptors could achieve better results with respect to the highly anti-EGRF specific panitumumab.

We evaluated the responses of the CRC cell lines to afatinib (AFA), a small-molecule receptor tyrosine kinase inhibitor (TKI), which irreversibly blocks signaling activity from all ErbB-family homo- and heterodimeric receptors [22]. Afatinib is currently approved for first-line treatment of patients with EGFR-mutation-positive lung cancer [23].

**Fig. 3:**
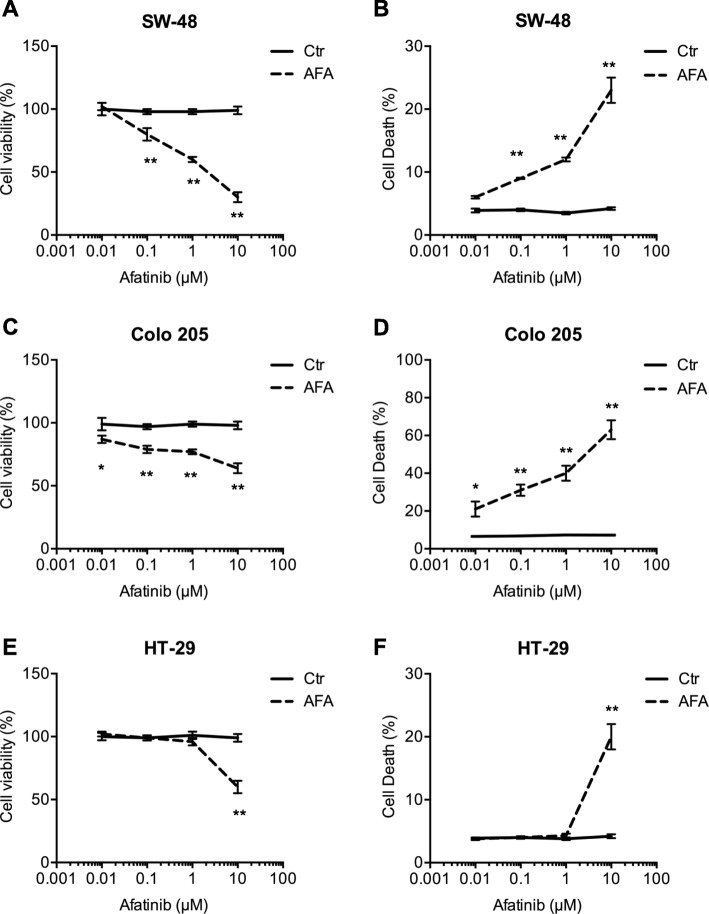
BRAF^V600E^ CRC cells respond to different doses of the pan-ErbB-family inhibitor afatinib. Cells were exposed to log10-escalating doses of afatinib (AFA-final concentration range: 0.01 μM to 10 μM) for 48 h, harvested, and assayed for (**a**, **c**, **e**) cell viability and (**b**, **d**, **f**) cell death, as described in Fig. 1. Results shown are means ± SD of three independent experiments. Statistics (two-way ANOVA for multiple comparisons): *p < 0.05, **p < 0.01 vs. CTRL (untreated cells)

Viability and death rates were assessed in all three CRC cell lines after exposure to AFA at concentrations ranging from 0.01 μM to 10 μM (log10 escalations). As expected SW-48 cells, which harbor an EGFR mutation targeted by afatinib (i.e., p. G719S), were sensitive to low doses of the drug (0.1 and 1 μM) (Fig. 3a, b). The responses of the BRAF^V600E^ cell lines—both of which are EGFR-wildtype—differed: in Colo205 cells significant changes in both viability and death rates were already evident after exposure to the lowest concentration tested (0.01 μM) (Fig. 3c and d), whereas HT-29 cells were significantly affected only by the highest concentration used (10 μM) (Fig. 3e and f).

### Vemurafenib and afatinib produce additive growth-limiting effects in CRC BRAF^V600E^ cell lines

We then wanted to investigate if the combination of vemurafenib with afatinib, which is able to target ErbB2 and ErbB4 receptors in addition to EGFR, could achieve better results than the combination of vemurafenib and panitumumab.

We then exposed the cells to vemurafenib and afatinib— the latter at doses of 1 μM or 10 μM (VEM + AFA1 and VEM + AFA10, respectively).

**Fig. 4:**
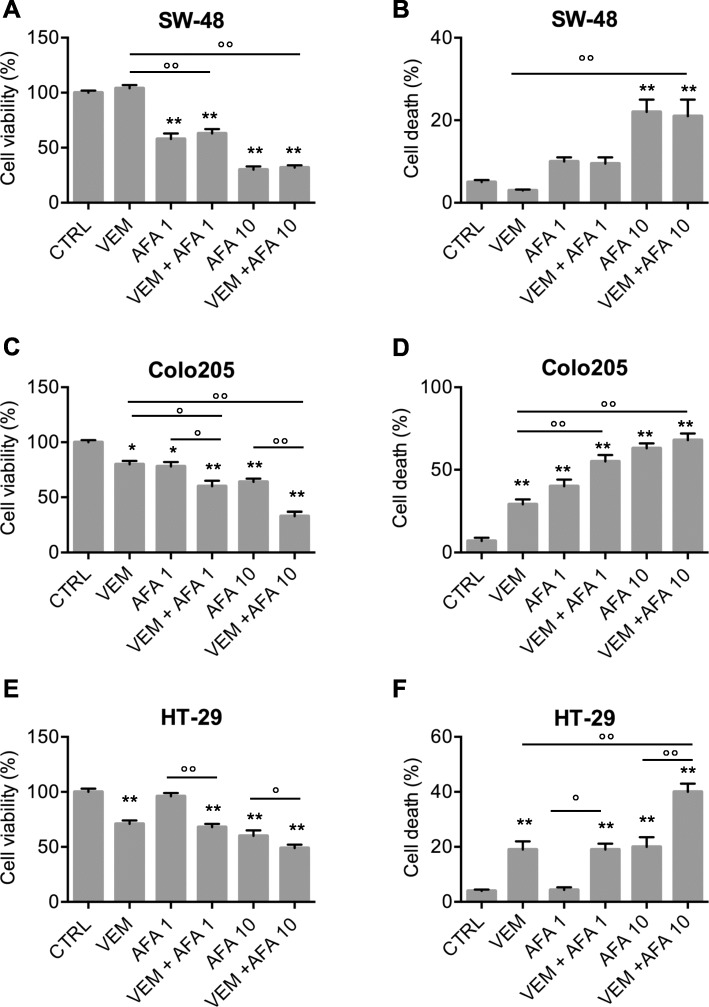
Vemurafenib and afatinib produce additive growth-limiting effects in CRC BRAF^V600E^ cell lines. Cells were exposed to VEM (3 μM), AFA (1 μM or 10 μM), or both (VEM + AFA1, VEM + AFA10, respectively) for 48 h, harvested, and assayed for (**a**, **c**, **e**) cell viability and (**b**, **d**, **f**) cell death. Results are means ± SD of three independent experiments. Statistics (two-way ANOVA for multiple comparisons): *p < 0.05, ** *p* < 0.001 vs. CTRL (untreated cells); ° p < 0.05, °° p < 0.01 vs. cells treated with the indicated drug or drug combo (horizontal bars)

As shown in Fig. 4a and b, the addition of vemurafenib did not enhance the effect produced in SW-48 cells by exposure to AFA1 or AFA10 alone, which was consistent with these cells’ unresponsiveness to the BRAF inhibitor alone (Fig. 1). In the BRAF^V600E^ lines (Colo205 and HT-29), the two drugs produced additive growth-limiting effects that were afatinib dose-dependent (Fig. 4c-f). In the Colo205 cells, which responded to both VEM and AFA when used alone (in terms of both decreased viability and increased death rates), the response to the combination VEM + AFA was stronger than those achieved with VEM + PAN (Fig. 2c and d), and these effects were even more evident when the higher dose of AFA was used (VEM + AFA10). As for the HT-29 cells, which were not significantly affected by VEM-PAN, the VEM-AFA1 combination was also effective, but the level of efficacy observed in Colo205 cells could be achieved only with the higher dose of AFA (VEM + AFA10).

### ErbB2/HER2/Neu is differentially expressed in untreated BRAF^V600E^ CRC cell lines

To explore possible mechanisms underlying the differential responses to the inhibition of the ErbB receptor family members of the BRAF^V600E^ cell lines, we analyzed their expression levels of ErbB2/HER2/Neu, which is targeted by AFA but not by PAN. Unlike EGFR, ErbB2 is activated not by ligand binding, but as a result of its heterodimerization with other ErbB family members. Owing to ErbB2’s constitutive kinase activity, EGFR/ErbB2 heterodimers are more active than EGFR homodimers, but this interaction is limited by the relatively low-level of ErbB2 expression in normal cells [24]. Mutations or amplifications in one of the four ERBB family genes are present in 22 out of 165 (13%) non-hypermutated and 16 out of 30 (53%) hyper-mutated cases [2]. Interestingly, activating mutations and amplifications of ErbB2 account for 7% of cases (Cancer Genome Atlas Network) and patients with HER2-amplified metastatic CRC are less likely to respond to anti-EGFR therapy [25].

**Fig. 5:**
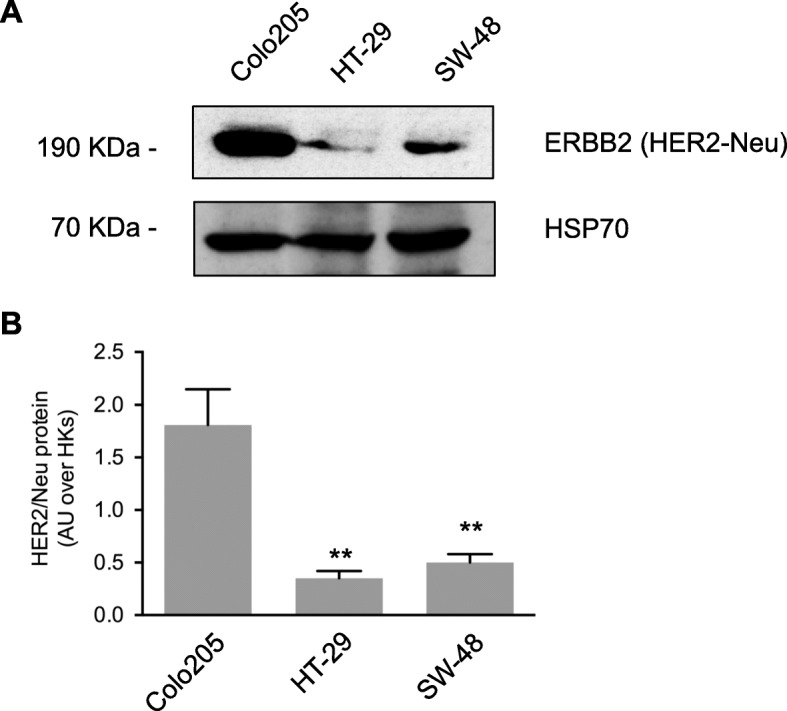
ErbB2/HER2/Neu is differentially expressed in untreated BRAF ^V600E^ CRC cell lines. **a** Representative immunoblots with (**b**) densitometric quantification of band intensities show ErbB2/HER2/Neu) protein levels in the three cell lines. (Loading control: HSP70) Bars in (B) represent means ± SD of three independent experiments. Statistics (test used): ** p < 0.01 vs. Colo205 cells

As shown in Fig. 5a and b, ErbB2 expression in Colo205 cells was significantly higher than that found in HT-29 (or in the BRAFwt SW-48 cells). This molecular feature could conceivably explain the high sensitivity of Colo205 cells to afatinib shown in Figs. 3 and 4. These findings suggest that ErbB2 expression levels in BRAF^V600E^ CRC cells are an important predictor of their responsiveness to ErbB blockade, alone or with BRAF inhibition. Patients whose tumors express high levels of ErbB2 are likely to be sensitive to afatinib monotherapy or combined treatment with afatinib and vemurafenib.

Indeed, in clinical settings, vemurafenib alone proves to be insufficient in CRC tumors owing in many cases to feedback-mediated reactivation of RAS. ErbB2 (HER2-Neu) has been identified as a possible mediator of this feedback. Our experiments showed that the small molecule pan-ErbB family inhibitor afatinib effectively reduces the viability of CRC cells. Moreover, responses to afatinib treatment were heterogeneous. Based on the ErbB2 protein levels we demonstrated in the cell lines we used, it seems reasonable to speculate that high-level expression of ErbB2 might predict an effective response to AFA at lower doses. We therefore propose that combined treatment with vemurafenib and afatinib could be used for BRAF^V600E^ CRCs, after confirming these in vitro results by testing the association in randomized clinical trials. Assessment of ErbB2 (HER2-Neu) expression levels in these tumors should also be used to inform treatment decision-making.

## Discussion

CRC includes different categories of tumors based on their specific mutational spectra and molecular phenotype, driving distinct oncogenic signaling pathways. The main goal of molecular oncology is the development of personalized medicine, and this is particularly urgent for BRAF mutant CRC patients since effective therapies are still lacking. Indeed, the inhibition of the BRAF^V600E^ oncoprotein by the small-molecule drug vemurafenib (VEM), which is highly effective in melanoma [26], showed a very limited response in these patients [27]. In BRAF mutant CRC, some studies have indicated that EGFR reactivation contributes to insensitivity to vemurafenib. In this context, in vivo studies showed that BRAF mutant patients could be responsive to association of BRAFi and EGFRi [3], mostly if associated with chemotherapy [28, 29]. Indeed, the combination of vemurafenib, irinotecan and panitumumab/cetuximab has now been included in the National Comprehensive Cancer Network (NCCN) guidelines for the second line therapy of BRAF mutant CRC patients [29, 30]. Similarly, other combinatorial strategies including BRAFi and EGFRi paired with MEK inhibitors have shown improvement in patients’ survival in randomized clinical trials and have been included in consensus guidelines [29–32]. Our study aimed to explore the effectiveness of different targeted drugs either alone or in combination in BRAF^V600E^ cell lines, with the aim to shed light on the possible mechanisms driving resistance to biological therapy in BRAF mutant patients. We found that the BRAF^V600E^ cell lines we tested differed markedly in their sensitivity to short-term exposure to the BRAF inhibitor VEM, as reflected by assays of cell viability and cell death. All cell lines were unresponsive to the anti-EGFR blockade with panitumumab, whereas all displayed sensitivity to afatinib (Table 3), which covalently binds to and blocks HER2 and HER4, in addition to EGFR, a dual receptor tyrosine kinase inhibitor for the treatment of solid tumors [33, 34]. Notably, combination strategies VEM + AFA at low dose achieved good response in Colo205 BRAF^V600E^ mutated cell lines (Table 3). Looking for molecular aspects that could explain the different responsiveness of the BRAF mut cells analyzed, we showed that Colo205 cells, that express higher levels of ErbB2 (HER2-Neu) in respect to the other cells, were the cells that presented the best impairment after afatinib treatment, even at low dose. We decided to consider the HER2-Neu molecular aspect in our cells since it has been described that human CRC are positive for HER2 staining [35] and that activating mutations and amplifications of ErbB2 account for 7% of cases (Cancer Genome Atlas Network). Moreover, HER2-Neu has been shown to be a promising prognostic and predictive target, recently investigated in several CRC clinical trials [29, 36–38]. Indeed, anti-HER2 targeted therapies have been included in clinical guidelines [30]. A very recent paper proposed the use of ErbB protein levels as biomarker for selection and stratifications of CRC patients [39]. Our data support the importance of ErbB2 evaluation, by the immunohistochemical staining of tumor samples after surgery, as a new molecular aspect in CRC mutated patients for personalized therapy selection.

**Table 3:** CRC cell lines response to the different tested drugs

Cell Line	Venurafenib	Afatinib	Panitumumab	Venurafenib+. Panitumumab	Venurafenib+ Afatinib
SW-48	–	+ (L)	–	–	- +
Colo-205	+	+ (L)	–	+ +	+ + (L)
HT29	+	+ (H)	–	+ −	+ + (H)

Legend: *L* Low doses of Afatinib *(0.01, 0.1, 1 μM)*, *H* High dose of Afatinib (10 μM)

We suggest screening tumors for the HER2-Neu expression since its high levels could be considered as positive predictive factor of treatment response using afatinib or using afatinib+vemurafenib.

## Conclusion

Our work presents new molecular aspects of BRAF mutated CRC cells which can occur in resistant patients and support the notion that, besides the specific BRAF^V600E^ mutation, other signaling pathway activations could be responsible for therapy failure. Therefore, BRAF mutant patients should not be considered as having a unique underlying biology but heterogeneous paths [20], that may be identified and exploited for effective personalized targeted therapies [40].

## Data Availability

The datasets used and/or analyzed during the current study are available from the corresponding author on reasonable request.

## Abbreviations

AFA: Afatinib
APC: Adenomatous polyposis coli
CRC: Colo-rectal Cancer
EGFR: Epidermal Growth Factor Receptor
ErbB2: Receptor tyrosine-protin kinase erbB-2
ERK: Extracellular signal–regulated kinases
HSP70: Heat shock protein 70
KRAS: Kirsten RAt Sarcoma
MAPK: Mitogen-activated protein kinase
PAN: Panitumumab
PCR: Polymerase chain reaction
PIK3CA: Phosphatidylinositol-4,5-bisphosphate 3-kinase catalytic unit
PTEN: Phosphatidylinositol-3,4,5-trisphosphate 3-phosphatase protein
RTKs: Receptor Tyrosine Kinsae
TKI: Tyrosine Kinase Inhibitor
TP53: Tumor Protein p53
VEM: Vemurafenib
WT: Wild type
